# Development of Virtual Reality Health Literacy: Delphi Expert Consensus Study

**DOI:** 10.2196/85842

**Published:** 2026-06-24

**Authors:** Junghee Yoon, Mangyeong Lee, Dokyoon Kim, Joungwon Park, Su jin Kim, Jiyoon Han, Juhee Cho

**Affiliations:** 1 Department of Clinical Research Design and Evaluation, Samsung Advanced Institute for Health Sciences & Technology, Sungkyunkwan University, 115 Irwon-ro, Gangnam-gu, Seoul, 06355, Republic of Korea, 82 2-3410-1448, 82 2-3410-6639; 2 Institute for Quality of Life in Cancer, Samsung Medical Center, Seoul, Republic of Korea; 3 Department of Digital Health, Samsung Advanced Institute for Health Sciences & Technology, Sungkyunkwan University, Seoul, Republic of Korea; 4 Center for Clinical Epidemiology, Samsung Medical Center, Seoul, Republic of Korea; 5 AI Research Center, Research Institute for Future Medicine, Samsung Medical Center, Seoul, Republic of Korea; 6 Division of Social Work, Samsung Medical Center, Seoul, Republic of Korea; 7 Cancer Education Center, Samsung Medical Center, Seoul, Republic of Korea

**Keywords:** virtual reality, VR, health literacy, Delphi study, domains

## Abstract

**Background:**

Virtual reality (VR) is a promising tool in health care, offering immersive and interactive environments that can enhance patient education, rehabilitation, and mental health interventions. However, effective patient engagement with head-mounted display (HMD)–based immersive VR depends on a combination of functional competencies and readiness-related determinants that have not yet been systematically defined.

**Objective:**

This study aimed to conceptualize an initial framework of VR health literacy, focused on HMD-based immersive VR in clinical settings, and to achieve expert consensus on its definition, domains, and subdomains.

**Methods:**

A 3-phase modified Delphi study was conducted between January and April 2024, including a literature review in MEDLINE (via PubMed) and Embase (2017-2023) informed by scoping review methodology, a multidisciplinary expert panel formation, and 2 online survey rounds, in which panelists rated each subdomain on a 4-point necessity scale and provided open-ended feedback, with 15 experts from the health care, VR, and health literacy fields. Consensus was defined using IQRs and agreement thresholds; items with moderate consensus were further evaluated through structured internal deliberation.

**Results:**

A total of 15 experts participated in Round 1, and 13 continued to Round 2 (retention rate, 87%). An initial structure of 7 candidate domains with 23 subdomains was iteratively refined across the two rounds based on consensus levels, expert panel feedback, and internal deliberation, with consensus thresholds applied as guiding criteria rather than automatic exclusion rules; subdomains with moderate consensus were further evaluated through structured internal deliberation to determine theoretical necessity within the framework. The final framework comprised 5 domains and 14 subdomains: performance expectancy (perceived usefulness of VR for health management; expectations of future VR benefits); effort expectancy (perceived immersion or embodiment; perceived interactivity and responsiveness; understanding of VR-related terms); facilitating conditions (access to VR devices and platforms; digital knowledge and confidence; technical proficiency with VR devices; digital self-efficacy); attitudes toward VR (awareness of VR in health contexts; interest in VR technology; problem-solving ability using VR content); and behavioral intention (intention to use VR technology or services; willingness to engage with VR for health).

**Conclusions:**

This study presents an initial consensus-based framework of VR health literacy for HMD-based immersive VR in clinical settings. Developed through a multidisciplinary Delphi process, the framework combines operational competencies with engagement-related determinants to provide both theoretical clarity and practical use, offering guidance for clinicians, educators, and policymakers to design and implement VR interventions that are accessible, equitable, and effective in health care contexts.

## Introduction

Virtual reality (VR) is a technology that generates an artificial, 3-dimensional environment using computer systems, allowing users to experience simulations that resemble the real world through displays, ranging from isolated screens to wearable options like head-mounted displays (HMDs) [1,2]. By engaging multiple senses, VR offers immersive experiences with realistic 360-degree visuals, real-time interaction, and artificial intelligence–driven personalization through intelligent avatars, enabling active user participation and creating a dynamic virtual environment unlike traditional media [3]. In this study, the term “VR” specifically refers to immersive VR systems delivered through HMD devices. While extended reality (XR) encompasses a broader spectrum, including augmented reality and mixed reality, the present framework focused on immersive HMD-based VR, as this modality currently represents the most widely implemented form of VR in clinical settings and presents distinct cognitive demands and safety considerations for users [4]. VR technology is a groundbreaking tool in health care interventions, offering immersive and interactive environments that enable patients to actively engage in their health management [5,6]. By simulating real-life scenarios, enhancing experiential learning, and providing tailored interventions such as multisensory high-end systems and game-based systems, VR has demonstrated promise in areas such as rehabilitation, mental health, and chronic disease management [7-9]. The capability of VR to deliver highly personalized and immersive experiences has been recognized as a pivotal advancement in the domain of patient-centered interventions [10].

The adoption of VR in health care faced several challenges, including the lack of a comprehensive framework to support patient engagement. The unique skills required for effective VR use, such as navigation and content interpretation, were often underdeveloped, limiting its accessibility and increasing inequalities in its use. A previous study highlighted that VR interaction capabilities differ significantly based on literacy and technological familiarity, with nonliterate users facing challenges in navigation, visual comprehension, and complex task execution [11]. The clinical adoption of VR has been reported to face obstacles such as limited VR knowledge, unfavorable attitudes, reluctance to use the technology, and usability difficulties, largely stemming from insufficient VR-related literacy and experience [5,12,13]. These challenges highlighted the urgent need to define and develop VR health literacy as a critical framework.

To the best of our knowledge, VR health literacy remains an unfamiliar concept within the existing literature. Therefore, developing this concept is essential to ensure that individuals can benefit from VR technologies regardless of their prior experience or technological proficiency [14,15]. Previous studies predominantly focused on usability or feasibility evaluations of VR use for patients or health-related applications among individuals with limited literacy skills [8,16,17].

To address this gap, we aimed to conceptualize VR health literacy as a framework to enhance individuals’ capacity to engage with VR-based health care interventions equitably and effectively. Therefore, we conducted a modified Delphi study to systematically establish the scope and components of VR health literacy, achieving consensus on the definition and identifying the essential subdomains.

## Methods

### Study Design

#### Overview of the Delphi Process

**Figure 1.**
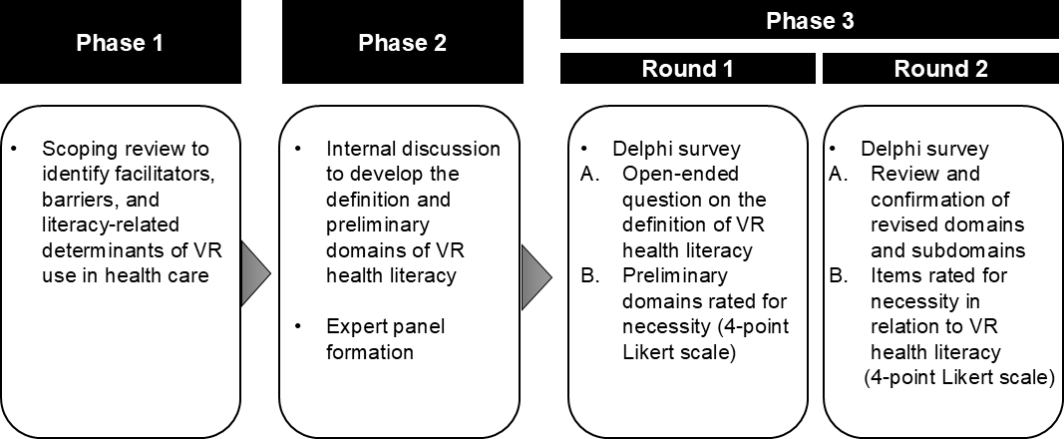
Overview of the 3-phase modified Delphi process used to develop the virtual reality (VR) health literacy framework. Phase 1 involved a scoping review to identify facilitators, barriers, and literacy-related determinants of VR use in health care. Phase 2 comprised internal expert discussions to develop the working definition of VR health literacy, generate preliminary domains, and form the multidisciplinary expert panel. Phase 3 consisted of 2 Delphi survey rounds. In Round 1, experts provided open-ended definitions of VR health literacy and rated preliminary domains for necessity using a 4-point Likert scale. In Round 2, experts reviewed and confirmed revised domains and subdomains and rated their necessity in relation to VR health literacy using a 4-point Likert scale.

We conducted a 3-phase modified Delphi study to develop and achieve consensus on a conceptual framework for VR health literacy. This design is suitable for identifying VR-specific factors within the framework of digital health literacy to advance the effective adoption and use of VR in clinical practice [18]. It enables expert consensus through iterative surveys and controlled feedback and offers a systematic framework for addressing complex challenges (Figure 1). The Delphi process involved 2 rounds of online surveys: the first survey was conducted between January 27 and February 8, 2024, and the second between April 18 and 25, 2024. Surveys were distributed via email, and the study adhered to the principles outlined in the Guidance on Conducting and Reporting Delphi Studies [19].

#### Phase 1. Literature Review Informed by Scoping Review Methodology for Item Generation

The initial phase consisted of a literature review informed by scoping review methodology to identify facilitators and barriers to VR use in health care and to explore literacy components associated with its application. An initial pilot search combining “virtual reality” and “health literacy” yielded a limited number of studies, most of which examined VR as a health literacy intervention rather than exploring literacy-related competencies required for VR engagement. Therefore, the search scope was expanded to include studies addressing usability, implementation barriers, and facilitators of VR use. PubMed and Embase were searched for studies published from January 2017 to December 2023, combining VR-related terms (eg, “virtual reality,” “VR”) with engagement-related terms (eg, literacy, usability, barriers, facilitators, adoption, implementation) and health-related terms (eg, health, health care, clinical); the full Boolean search strings are provided in Multimedia Appendix 1. The combined search yielded approximately 210 records after deduplication. Studies examining user engagement, implementation, or experiential aspects of health care VR in adult patients were included; records were excluded if they addressed non-VR digital technologies, pediatric or adolescent populations, provider-training contexts, or lacked a clear clinical application context. Following title and abstract screening against these criteria, approximately 30 studies were retained for closer reading, and approximately 15 of these were examined in depth as primary sources for factor extraction. Records were reviewed collaboratively by 3 research team members through iterative discussion until consensus was reached on each study’s conceptual relevance, and the extracted factors informed the initial domain and subdomain generation for the Delphi rounds.

This literature review revealed that expectancy-related constructs consistently emerged as determinants of VR adoption and sustained use in health care contexts. Traditional health literacy frameworks predominantly focus on cognitive and functional skills required to access, understand, and apply health information [20]. However, engagement with technology-mediated health interventions involves an additional layer of evaluation: users must appraise not only the health content itself but also the technological medium through which this content is delivered, including its usability and perceived benefit. In immersive VR contexts, these evaluations directly influence whether users initiate and sustain engagement with the system, thereby conditioning the enactment of literacy-related competencies in practice. This gap between traditional health literacy conceptualizations and the requirements of VR-based interventions informed the incorporation of expectancy-related constructs as engagement-enabling components within our framework.

#### Phase 2. Expert Panel Formation and Item Development

An expert panel was convened with professionals representing health care, VR technology, and health literacy. Eligibility criteria required at least 1 prior experience in VR-related research or educational activities and included (1) health care professionals (eg, physicians, nurses, researchers), (2) experts in health care or health communication, and (3) specialists in digital health literacy research or interventions. The 1-year threshold for VR-related experience was determined after extensive internal discussion, balancing the need for practical exposure to immersive VR implementation against the limited availability of professionals with combined expertise in VR technology and health literacy in this emerging field. The second phase focused on developing the initial survey instrument for the Delphi study. Based on findings from the scoping review, an internal expert panel consisting of 2 digital health experts, 1 VR specialist, and 2 patient education researchers was convened.

Furthermore, to ensure integration of health literacy theory with VR-specific technological expertise, the research team was deliberately composed to intentionally include members who would bring complementary disciplinary perspectives. The core team comprised investigators with extensive backgrounds in VR technology development and clinical implementation (including 1 member with over 20 years of experience in immersive technology design for health care and education), as well as investigators with established expertise in health literacy research, including the development of nationally validated health literacy assessment instruments and empirical investigations of digital health literacy across diverse populations. This multidisciplinary composition functioned as a theoretical and methodological safeguard, informing all stages of the study methodology. By using a consensus-based process, this panel identified essential factors, proposed preliminary domains, and subsequently generated, prioritized, and refined the corresponding survey items. Each item was carefully reviewed to ensure its relevance, clarity, and alignment with the core aspects of VR health literacy before proceeding to the first Delphi round. Item generation was conducted collaboratively, with each preliminary item reviewed by both health literacy scholars and VR technology experts to balance theoretical grounding with medium-specific competencies. Throughout the Delphi process, panel feedback and emerging consensus were continuously evaluated by team members from both disciplinary backgrounds to mitigate potential disciplinary bias and maintain alignment between health literacy theory and VR implementation considerations.

#### Phase 3. Delphi Rounds

Two rounds of Delphi surveys were conducted to refine the definition and domains of VR health literacy and to assess the relevance of its constituent domains and subdomains.

In Round 1 (R1), 2 components were included to capture initial expert input and prioritize the necessity of proposed domains. First, participants provided open-ended definitions of VR health literacy, ensuring diverse perspectives for conceptualization. Items related to expert-identified needs for integrating VR in health care management were rated using an agreement scale ranging from “strongly disagree” to “strongly agree.” In contrast, items assessing VR health literacy domains and subdomains were evaluated using a necessity scale ranging from “not necessary” to “very necessary.” Second, participants were presented with a list of detailed domains related to VR health literacy and asked to rate their necessity on a 4-point Likert scale ranging from not necessary to very necessary*.*

In Round 2 (R2), the survey focused on achieving consensus on the refined framework. Participants reviewed the revised domains and subdomains from R1 and evaluated the necessity of each item using a 4-point Likert scale (“not necessary at all” to “very necessary”). Additionally, participants were invited to provide qualitative feedback to suggest further refinement. In this round, participants were informed that the evaluation would focus specifically on HMD-based immersive VR systems. This clarification was provided because several R1 responses referenced broader XR modalities. Narrowing the scope ensured conceptual consistency and alignment with the modality most commonly implemented in clinical health care settings.

### Statistical Analysis

Descriptive statistics, including mean (SD), median (IQR), and agreement levels, were used to analyze the quantitative data from each Delphi round. Agreement was defined as the proportion of respondents who rated a statement as 3 or 4 on a 4-point Likert scale [18]. For the qualitative data, thematic analysis was applied to the open-ended responses to extract common themes related to the definition and components of VR health literacy.

The criteria for consensus were defined as follows: “high consensus” required an IQR ≤1 and agreement percentage ≥80%; “moderate consensus” required an IQR ≤1 and agreement percentage between 50% and 79%; and “low consensus” was identified by an IQR >1 and agreement percentage <50%. The dataset was analyzed using R software (version 4.2.3; R Foundation for Statistical Computing). Consensus thresholds were applied as guiding criteria rather than automatic exclusion rules. Subdomains with moderate consensus were further evaluated through structured internal deliberation to determine theoretical necessity within the framework.

### Ethical Considerations

This study was approved by the institutional review board of Samsung Medical Center (approval number SMC 2023-12-070-011). All participants provided informed consent prior to their involvement in the study. Participant privacy and confidentiality were strictly maintained throughout the study. All collected data were deidentified and stored securely, and only the research team had access to the study data. The study was conducted in accordance with applicable ethical requirements and relevant regulations concerning the protection of personal information, privacy, and human rights. Participants received compensation of KRW 50,000 (approximately US $37) per round for their participation in the 2-round Delphi process (total KRW 100,000, approximately US $73).

## Results

A total of 15 experts participated in R1, with 13 continuing to R2, as summarized in Table 1. The panel included various health care professionals, including hospital researchers and professors (n=5), IT developers (n=5), and researchers from industry promotion foundations, public institutions, or government organizations (n=5). While some experts had relatively limited VR-related experience, averaging 1 year, the panel also included senior participants with up to 12 years of VR expertise and over 25 years of experience in health care research. This diversity provided a balanced perspective; however, we acknowledge the limited pool of dual-expertise professionals as a study limitation. Of the 15 participants in R1, 13 (87%) continued to R2, reflecting a high retention rate for Delphi studies.

**Table 1.** Descriptive characteristics of expert panel participants (N=15).

Number	Career	VR^a^-related experience (y)	Health care (y)	Education and research (y)	Health literacy experience (y)
1	Researcher	1	0	4	0
2	IT developer	1	0	0	0
3	Industry promotion foundation researcher	10	0	0	0
4	IT developer^b^	12	9	14	5
5	Hospital researcher	2	6	12	0
6	Hospital researcher	2	20	3	0
7	Industry promotion foundation researcher	3	0	12	0
8	IT developer	1	0	4	0
9	Hospital researcher	2	7	7	2
10	IT developer	1	0	0	0
11	Professor (doctor)^b^	5	25	24	5
12	Industry promotion foundation researcher	4	10	10	4
13	Research director	1	0	10	0
14	Hospital researcher	3	1	15	10
15	IT developer	1	2	0	0

a VR: virtual reality.

b Participated only in Round 1.

In R1, experts evaluated the need for VR applications in health care (Table 2). The highest consensus included VR-based content development research (mean 3.9, IQR 0, Q1-Q3 4-4, agreement 100%), simulations for patient and caregiver education (mean 3.9, IQR 0, Q1-Q3 4-4, agreement 100%), psychological therapy applications (mean 3.8, IQR 0, Q1-Q3 4-4, agreement 100%), and student or health care professional education (mean 3.8, IQR 0, Q1-Q3 4-4, agreement 100%). In contrast, areas related to direct medical care and treatment received lower scores, including diagnostic assistance (mean 2.7, IQR 1, Q1-Q3 2-3, agreement 60%), surgical support (mean 2.6, IQR 1, Q1-Q3 2-3, agreement 53.3%), prognosis monitoring and survivorship care (mean 2.8, IQR 1, Q1-Q3 2-3, agreement 64.3%), and symptom management, such as pain reduction (mean 2.9, IQR 2, Q1-Q3 2-4, agreement 66.7%).

**Table 2.** Expert-identified needs for integrating virtual reality in health care management and clinical practice^a^.

Domains and subdomains	Mean (SD)	Median	IQR (Q1-Q3)	Agreement (%)
Medical care				
Diagnostic assistance	2.7 (0.9)	3	1 (2-3)	60
Treatment				
Surgical support	2.6 (1.0)	3	1 (2-3)	53.3
Rehabilitation therapy	3.5 (0.5)	4	1 (3-4)	100
Psychological therapy (eg, trauma)	3.8 (0.4)	4	0 (4-4)	100
Symptom management (eg, pain reduction)	2.9 (1.1)	3	2 (2-4)	66.7
Cognitive training (eg, dementia prevention)	3.6 (0.5)	4	1 (3-4)	100
Prognosis monitoring and survivorship care (eg, cancer survivors)	2.8 (0.9)	3	1 (2-3)	64.3
Research				
VR-based^b^ content development research	3.9 (0.3)	4	0 (4-4)	100
Intervention efficacy studies	3.6 (0.5)	4	1 (3-4)	100
Patient or caregiver education				
Simulation (eg, emergency response)	3.9 (0.3)	4	0 (4-4)	100
Spatial orientation and navigation guidance	3.3 (0.7)	3	1 (3-4)	86.7
General public education				
Health promotion and disease prevention	3.1 (0.8)	3	1.5 (2.5-4)	73.3
Behavioral simulation (eg, exercise methods, healthy eating)	3.5 (0.7)	4	1 (3-4)	92.9
Student or health care professional education				
Clinical simulation (eg, surgery, CPR^c^)	3.8 (0.4)	4	0 (4-4)	100
Staff education				
Safety training simulation (eg, fire response)	3.7 (0.5)	4	1 (3-4)	100

a Ratings based on a 4-point agreement scale (1=strongly disagree; 4=strongly agree).

b VR: virtual reality.

c CPR: cardiopulmonary resuscitation.

As part of Delphi R1, experts were asked to provide open-ended definitions of VR health literacy. A diverse range of conceptual interpretations reflecting technical, cognitive, and practical dimensions of VR use in health contexts is summarized in Table S1 in Multimedia Appendix 2. Following iterative review and conceptual refinement of expert input, we defined VR health literacy as “the competency to acquire, understand, use, and manage health information and data through VR devices and services, enabling individuals to effectively engage with health-related content, promote health behaviors, and support health management.” This definition reflected a deliberate conceptual positioning. While VR-specific technological characteristics were considered during refinement, the framework ultimately positioned VR health literacy as health literacy applied within immersive VR contexts, with VR-specific competencies operationalized through the identified domains. Further empirical work may inform refinement of VR-specific competencies in future iterations of the framework.

Table 3 summarizes the mean (SD), median (IQR), and agreement percentages across 2 Delphi rounds, highlighting the iterative refinement of subdomains. Through iterative refinement, the initial 7-domain candidate structure entering R1 was refined through 2 rounds of consensus into a final 5-domain framework: (1) performance expectancy, (2) effort expectancy, (3) social influence, (4) facilitating conditions, (5) attitudes toward VR, (6) VR information literacy, and (7) behavioral intention, with 23 subdomains initially proposed during R1. Following R1, 9 subdomains were removed. Subdomains with moderate consensus or lower were excluded after internal deliberation, ensuring that the final set of subdomains was relevant and robust. Despite achieving high consensus (mean 3.4, IQR 0.6, agreement 93.3%) in R1, the “anticipated participation in VR-based health services” subdomain was excluded after internal deliberation. Experts noted that the absence of an established VR-based health service infrastructure and the limited availability of such programs undermined the current feasibility of this subdomain. In contrast, 3 new subdomains, including “expectations of future VR benefits,” “intention to use VR technology/services,” and “willingness to engage with VR for health,” were added, resulting in 17 subdomains being evaluated in R2. The 3 newly added subdomains were developed based on feedback from the expert panel, internal discussions, and additional literature reviews. However, the “understanding of VR-related terms” subdomain (mean 3.8, IQR 0.5, agreement 73.3%), despite achieving only moderate consensus, was retained based on its perceived importance as determined during expert panel discussions.

In R2, experts demonstrated high levels of consensus across multiple subdomains, particularly those related to immersive experiences, such as “perceived immersion/embodiment,” user-system interaction, such as “perceived interactivity and responsiveness,” and “digital self-efficacy,” each reaching 100% agreement (Table 3). The inclusion of 2 behavioral intention subdomains, “intention to use VR” and “willingness to engage with VR technology/services,” was also validated with strong support, reinforcing the importance of motivational factors. Finally, we included these 3 moderated consensus subdomains, including “expectations of future VR benefits,” “understanding of VR-related terms,” and “digital knowledge and confidence,” in the final framework based on internal deliberations regarding their importance and relevance to VR health literacy.

**Table 3.** Descriptive statistics and agreement levels for each subdomain in Delphi Rounds 1 and 2^a^.

Domains	Round 1				Decision after Round 1	Round 2			
	Mean (SD)	Median	IQR^b^ (Q1-Q3)	Agreement (%)		Mean (SD)	Median	IQR (Q1-Q3)	Agreement (%)
Performance expectancy									
Perceived usefulness of VR^c^ for health management	3.3 (0.6)	3	1 (3-4)	93.3	No change	3.5 (0.9)	4	1 (3-4)	92.3
Anticipated participation in VR-based health services	3.4 (0.6)	3	1 (3-4)	93.3	Deleted	—^d^	—	—	—
Expectations of future VR benefits	—	—	—	—	Added	2.9 (0.8)	3	1 (2-3)	69.2
Effort expectancy									
Perceived immersion or embodiment	3.1 (0.8)	3	1 (3-4)	86.7	No change	3.7 (0.5)	4	1 (3-4)	100
Perceived interactivity and responsiveness	3 (0.8)	3	0.5 (3-3.5)	80	No change	3.5 (0.5)	4	1 (3-4)	100
Understanding of VR-related terms	3.8 (0.6)	3	0.5 (2.5-3)	73.3	No change	2.5 (1)	3	1 (2-3)	53.8
Social influence									
Support from peers or caregivers	2.6 (0.7)	3	1 (2-3)	60	Deleted	—	—	—	—
Recommendations or encouragement from others	2.5 (0.6)	2	1 (2-3)	40	Deleted	—	—	—	—
Health care professional endorsement	3.1 (0.8)	3	1.5 (2.5-4)	73.3	Deleted	—	—	—	—
Facilitating conditions									
Access to VR devices and platforms	3.1 (0.7)	3	1 (3-4)	80	No change	3.2 (0.4)	3	0.2 (3–3.2)	100
Digital knowledge and confidence	3.1 (0.7)	3	0.5 (3-3.5)	80	No change	2.9 (0.6)	3	0 (3-3)	76.9
Technical proficiency with VR devices	3 (0.7)	3	0 (3-3)	80	No change	3.3 (0.8)	3	1 (3-4)	84.6
Prior experience with VR (direct/indirect)	2.5 (0.7)	2	1 (2-3)	40	Deleted	—	—	—	—
Digital self-efficacy	3.1 (0.6)	3	0.5 (3-3.5)	86.7	No change	3.5 (0.5)	4	1 (3-4)	100
Attitudes toward VR									
Awareness of VR in health contexts	3.2 (0.6)	3	0.5 (3-3.5)	93.3	No change	3.4 (0.7)	3	1 (3-4)	92.3
Interest in VR technology	3.2 (0.7)	3	1 (3-4)	86.7	No change	3.1 (0.5)	3	0 (3-3)	92.3
VR information literacy									
Ability to acquire and process VR-based health info	2.5 (0.5)	2	1 (2-3)	46.7	Deleted	—	—	—	—
Evaluation of reliability, relevance, and technology	2.7 (0.7)	3	1 (2-3)	53.3	Deleted	—	—	—	—
Critical thinking about VR content and tools	2.7 (0.7)	3	1 (2-3)	53.3	Deleted	—	—	—	—
Problem-solving ability using VR content	3.1 (0.7)	3	0.5 (3-3.5)	80	No change	3.2 (0.9)	3	1 (3-4)	84.6
Behavioral intention									
Intention to use VR technology or services	—	—	—	—	Added	3.5 (0.9)	4	1 (3-4)	92.3
Willingness to engage with VR for health	—	—	—	—	Added	3.8 (0.4)	4	0 (4-4)	100
Sharing VR experiences with others	2.8 (0.8)	3	1 (2-3)	60	Deleted	—	—	—	—

a Seven candidate domains entered Round 1; the final framework comprises 5 domains and 14 subdomains. “Social influence” and “VR information literacy” did not reach consensus and were dissolved. “Problem-solving ability using VR content” was reassigned to “Attitudes toward VR.”

b The IQR is calculated by subtracting the first quartile (Q1) from the third quartile (Q3). "High consensus" required an IQR ≤1, agreement percentage ≥80%; "moderate consensus" required an IQR ≤1, agreement percentage between 50% and 79%; and "low consensus" was identified by an IQR >1, agreement percentage. Ratings based on a 4-point agreement scale (1=not necessary; 4=very necessary).

c VR: virtual reality.

d Not applicable.

In the refinement process, several subdomains were deleted, some newly added, and others reclassified (Table 4). Notably, the subdomain “problem-solving ability using VR content,” initially categorized under VR information literacy, was integrated into the domain “attitudes toward VR.” Experts noted that problem-solving reflects cognitive engagement and motivational attitudes toward VR use rather than technical literacy alone. Based on this iterative process, the final VR health literacy framework comprised 5 overarching domains and 14 subdomains, each representing essential competencies for engaging with VR in health contexts (Table 3). “Performance expectancy” included “perceived usefulness of VR for health management” and “expectations of future benefits” from its integration into health care. “Effort expectancy” encompassed perceptions of immersion, interactivity, and the ability to understand VR-related terminology. “Facilitating conditions” captured the practical enablers of VR use, such as access to devices and platforms, digital knowledge and confidence, technical proficiency, and digital self-efficacy. “Attitudes toward VR” reflected individuals’ awareness of VR applications in health and interest in VR technology, including their problem-solving capabilities. Lastly, “behavioral intention” was represented by individuals’ intention to use and willingness to engage in VR-based health interventions.

**Table 4.** Definitions of finalized subdomains of virtual reality health literacy.

Domains and subdomains	Definitions
Performance expectancy	
Perceived usefulness of VR^a^ for health management	The individual’s belief that VR technologies and services can generate meaningful value and improved outcomes in the context of health management
Expectations of future VR benefits	The degree to which individuals anticipate positive future outcomes or health-related advantages from the continued advancement and integration of VR technologies
Effort expectancy	
Perceived immersion or embodiment	The subjective sense of being physically and psychologically present in the virtual environment while using VR devices for health-related purposes
Perceived interactivity and responsiveness	The extent to which users perceive VR systems and content as interactive, responsive, and capable of enabling real-time engagement in a health context
Understanding of VR-related terms	The ability to comprehend basic terminology, concepts, and language necessary to navigate and use VR platforms and services effectively
Facilitating conditions	
Access to VR devices and platforms	The individual’s physical and digital accessibility to VR equipment, applications, and platforms applicable to health monitoring, education, or intervention
Digital knowledge and confidence	The foundational knowledge of digital technologies and the confidence to navigate digital tools, including those specific to VR, in health-related settings
Technical proficiency with VR devices	The practical ability to operate essential functions of VR devices and applications required for effective participation in digital health experiences
Digital self-efficacy	An individual’s belief in their own capacity to successfully engage with and perform tasks related to digital health, including VR-mediated interventions
Attitudes toward VR	
Awareness of VR in health contexts	Recognition of the presence, relevance, and potential applications of VR technology within medical, therapeutic, or public health settings
Interest in VR technology	The cognitive and emotional inclination to explore, learn about, and engage with VR technologies, especially as they pertain to health and well-being
Problem-solving ability using VR content	The capability to address or manage health-related challenges through the strategic use of VR content, tools, or simulations
Behavioral intention	
Intention to use VR technology or services	The expressed willingness and planned behavior to engage with VR-based tools and services as part of ongoing or future health practices
Willingness to engage with VR for health	The readiness to participate in VR-assisted health interventions or programs, including openness to adoption and behavioral engagement

a VR: virtual reality.

## Discussion

We conducted a modified Delphi study to establish the scope and components of VR health literacy, with the aim of developing a comprehensive framework and reaching consensus on its definition and essential subdomains. In this study, we defined VR health literacy as the competency to acquire, understand, use, and manage health information and data through VR devices and services, enabling individuals to engage with health-related content, promote health behaviors, and support health management. This definition builds on the integrated health literacy framework while extending it with VR-specific competencies such as immersion, interactivity, and technical proficiency unique to virtual environments. This study identified 5 core domains of VR health literacy, including (1) performance expectancy, (2) effort expectancy, (3) facilitating conditions, (4) attitudes toward VR, and (5) behavioral intention, as well as 14 subdomains.

Aligned with prior research, our expert panel recognized education, training, and mental health interventions as pivotal areas for VR use, underscoring the technology’s transformative potential in these domains [21,22]. However, in practice, the implementation of VR in health care is largely limited to basic applications, such as scenic distractions or the adaptation of pre-existing interventions [23,24]. While the adoption of VR in health care is steadily expanding, current applications are limited to basic functions, such as visualization and passive content delivery. Consequently, the broader potential of VR to support preventive health and behavioral improvement has not been fully realized in practice. This gap between technological potential and applied use highlights the need for a systematic framework to assess user competencies. For health care professionals who design and implement VR interventions, such insights are critical to ensuring that strategies are tailored to users’ cognitive, emotional, and contextual capacities, thereby maximizing both clinical effectiveness and educational impact.

In this study, we defined VR health literacy as an extension of digital health literacy that incorporates competencies unique to virtual environments, including immersion, interactivity, and technical proficiency. Early discussions of VR health literacy emphasized that VR is not only a technology but also a medium with its own emerging language and symbolic system [25]. Within this framework, VR health literacy includes the capacity to interpret and apply interaction cues such as gestures, avatars, and spatial representations in health care contexts. It is shaped by both users and content developers, underscoring its relevance for patients, clinicians, and educators. As the language of VR remains at an early stage, VR health literacy should be understood as a dynamic construct evolving alongside the expansion of immersive technologies in health care.

The Delphi results showed variation in consensus across domains. Subdomains related to performance and effort expectancy, particularly perceived usefulness, reached consistently high agreement. This finding is consistent with prior studies indicating that users value VR mainly for its immersive features and outcome-oriented benefits in health contexts [26]. Our Delphi findings underscore the fact that immersion and interactivity are central elements of VR health literacy, aligning with qualitative evidence [21] showing that these features enhance learner motivation and engagement. Notably, domains related to social influence were excluded. Although prior studies highlight the importance of peer and clinician recommendations for technology adoption [21], our Delphi panel gave these items lower priority. This may reflect the early stage of VR adoption in health care, where individual technical competencies are viewed as more urgent prerequisites than external influences. Future studies with broader public exposure to VR may yield different results. In addition, this contrast suggests that while assessment tools may appropriately focus on individual competencies, successful implementation in clinical practice will also depend on social and contextual drivers. Our results also underscore behavioral intention as a key determinant within VR health literacy. Consistent with prior research [27] in telerehabilitation, behavioral intention emerged as a key factor influencing adoption. These findings suggest that the successful integration of VR in health care relies not only on technical feasibility but also, importantly, on willingness to engage with and sustain the use of the technology. In contrast, despite acknowledging factors such as prior experience with VR, evaluation of reliability and relevance, critical thinking about VR content and tools, and sharing VR experiences as potentially relevant to VR health literacy, these subdomains were ultimately excluded due to low consensus within our Delphi panel. Although VR technologies are advancing rapidly, widespread engagement among the general public is still limited [28], with most exposure occurring through gaming rather than health-related applications. The exclusion of these domains from our Delphi consensus likely reflects this contextual backdrop of restricted exposure rather than conceptual irrelevance, indicating that their relevance should be revisited in future iterations of the framework as VR adoption expands beyond entertainment into health-related contexts.

In this study, expectancy- and intention-related constructs were included within the framework of VR health literacy to reflect the engagement-dependent nature of immersive VR environments. Contemporary developments in digital and eHealth literacy increasingly recognize that the enactment of literacy skills in technology-mediated contexts depends not only on operational ability but also on perceived capability, confidence, and readiness to engage [20,29]. HMD-based immersive VR requires active participation, spatial navigation, and sustained interaction for users to access health information. In such environments, literacy extends beyond the possession of technical skills to include the capacity to mobilize those skills within embodied and action-oriented contexts. Accordingly, performance expectancy, effort expectancy, and behavioral intention were conceptualized as engagement-enabling components that influence whether literacy-related competencies can be meaningfully enacted, rather than as direct substitutes for core cognitive literacy skills. Further field-based empirical validation is necessary to assess construct validity and clarify the functional interplay between motivational readiness and operational competencies in applied VR environments.

The 5 domains identified in this study constitute an initial operational framework of core competencies for the effective use of VR in health care. The framework was developed through a structured process integrating insights from health care professionals, IT developers, and public sector representatives, thereby reflecting clinical, technical, and policy perspectives. This multidisciplinary composition allowed diverse priorities to be reconciled, resulting in a model that combines theoretical clarity with practical applicability. The value of this framework extends beyond its theoretical structure. Developed through a multidisciplinary consensus process, it provides a practical tool for clinicians, educators, and policymakers to support the integration of VR into health care. The identified competencies may inform patient education curricula, guide assessment of readiness for VR-based interventions, and shape policy standards for safe and equitable implementation. VR health literacy was conceptualized not as proficiency confined within immersive environments, but as the capacity to engage with, interpret, and apply health information delivered through VR technologies across contexts. Although the framework does not include a standalone “transfer of knowledge” domain, the translation of VR-mediated information into real-world health understanding, decision-making, and communication is treated as a core functional outcome of literacy enactment. In health care contexts, immersive understanding is only meaningful as long as it informs subsequent health decisions, discussions with providers, and behavioral implementation beyond the virtual setting. This conceptualization positions VR health literacy as an applied construct oriented toward real-world health action, rather than as a measure of in-system proficiency alone. As such, the framework offers a foundation for translating VR health literacy from concept to practice [27,30].

In the present Delphi process, subdomains related to critical appraisal and information evaluation were carefully considered during item generation and expert review. Although these competencies are central to established health literacy frameworks, they did not reach consensus for inclusion in the final framework. This outcome was interpreted not as a theoretical devaluation of appraisal, but as a reflection of the current clinical context of immersive VR use, in which health content is typically curated and implemented within supervised health care settings. In such environments, functional readiness, embodied interaction, and engagement capacities may operate as prerequisites for subsequent evaluative competencies. Accordingly, the framework adopts a staged conceptualization of VR health literacy, emphasizing access and enactment capacities in its initial iteration while recognizing that appraisal-related competencies may become increasingly salient as VR platforms evolve toward more open and user-driven information ecosystems.

Furthermore, the present framework focuses specifically on HMD-based immersive VR systems rather than the broader spectrum of XR modalities. This staged conceptualization also informs our treatment of immersion-related competencies. Although immersion is fundamentally a property of immersive media, effective engagement in VR-based health interventions requires users to cognitively and behaviorally adapt to embodied and spatially dynamic environments. In this framework, perceived immersion or embodiment does not refer to the intensity or quality of subjective experience, but rather to the individual’s capacity to maintain spatial orientation, regulate embodied interaction, and integrate multisensory stimuli while processing health information. This distinction explicitly separates user experience quality from competency-related adaptation within immersive contexts, addressing the conceptual boundary between media properties and literacy-related capacities.

Regarding safety-related competencies, including recognition of cybersickness and maintenance of spatial awareness, we acknowledge their importance in immersive VR contexts. Although safety did not emerge as a distinct domain in the Delphi consensus, these elements were conceptually recognized during framework refinement and intentionally integrated across relevant domains given their cross-cutting nature. In interpreting these findings, we considered that many current health care VR applications are implemented in structured clinical environments with guided use and curated content, where safety-related awareness is often embedded within broader operational competencies. Accordingly, safety was positioned as an integral component of technical proficiency and facilitating conditions rather than defined as an independent construct in this initial framework. This positioning reflects a deliberate conceptual focus rather than an omission and may be revisited as VR applications expand into less supervised or nonclinical settings.

A key strength of this study is its innovative approach to establishing a foundational framework for the emerging field of VR health literacy. Nonetheless, this study has some limitations. First, the composition of the Delphi panel included variation in VR-specific experience levels among panel members, ranging from early-career professionals to senior experts, as well as a higher proportion of VR technology specialists relative to health literacy researchers. While this composition was intentionally designed to capture medium-specific competencies unique to immersive VR environments, differences in experience and disciplinary perspective may have influenced the prioritization of operational and engagement-related constructs. Future iterations of this framework would benefit from broader and more balanced panel representation, including patient advocates, health education researchers, and clinical practitioners, to further enhance conceptual balance and generalizability. In addition, as this study was conducted with experts based in Korea, cultural and systemic factors may have shaped the consensus. Future Delphi studies with multinational panels will be critical to validate and generalize the VR health literacy framework globally. Lastly, as VR technology continues to advance rapidly, the specific components of this framework may require updating over time. As a foundational study in this nascent field, the framework will naturally require further validation and refinement as the concept of VR health literacy evolves and its application in health care settings expands.

In conclusion, this study establishes an initial, consensus-based framework for VR health literacy. As VR technology approaches widespread clinical integration, this framework addresses the urgent need to enhance patient-centered competencies and align technological capabilities with clinical needs. It serves as both a theoretical model and a practical roadmap for researchers, clinicians, and developers to ensure that VR is implemented equitably and effectively. As immersive VR continues to evolve from supervised clinical implementation toward more autonomous and home-based use, domains that did not reach consensus in the present Delphi process—most notably critical appraisal of VR content and safety-related competencies such as recognition of cybersickness and maintenance of spatial awareness—are likely to gain increasing salience and warrant dedicated empirical investigation in future iterations of the framework. Ultimately, fostering VR health literacy is pivotal in unlocking the full potential of VR to transform health care, and this study provides a foundational step in that critical journey.

## Supplementary material

10.2196/85842 Multimedia Appendix 1 Full Boolean search strategies.

10.2196/85842 Multimedia Appendix 2 Expert perspectives on definition of virtual reality (VR) health literacy.

## Abbreviations

HMD: head-mounted display
R1: Round 1
R2: Round 2
VR: virtual reality
XR: extended reality
