# Supplementary material for: Development of Virtual Reality Health Literacy: Delphi Expert Consensus Study
Source: J Med Internet Res. 2026 Jun 24;28:e85842. doi: 10.2196/85842 (PMC13293473; doi:10.2196/85842)
Supplement: Multimedia Appendix 1 [file jmir-v28-e85842-s001.docx]

**Supplementary Material S1. Full Boolean Search Strategies**

| PubMed ("virtual reality"[MeSH Terms] OR "virtual reality"[Title/Abstract] OR "VR"[Title/Abstract]) AND ("health"[Title/Abstract] OR "healthcare"[Title/Abstract] OR "clinical"[Title/Abstract] OR "medical"[Title/Abstract]) AND (literac*[Title] OR barrier*[Title] OR facilitator*[Title] OR "user experience"[Title] OR "health literacy"[Title] OR "digital literacy"[Title] OR usability[Title] OR adoption[Title] OR engagement[Title] OR implementation[Title]) AND ("2017/01/01"[Date - Publication] : "2023/12/31"[Date - Publication])  Embase ('virtual reality'/exp OR 'virtual reality':ti,ab OR VR:ti,ab) AND (health:ti,ab OR healthcare:ti,ab OR clinical:ti,ab OR medical:ti,ab) AND (literac*:ti OR barrier*:ti OR facilitator*:ti OR 'user experience':ti OR 'health literacy':ti OR 'digital literacy':ti OR usability:ti OR adoption:ti OR engagement:ti OR implementation:ti) AND [2017-2023]/py |
| --- |
