# Supplementary material for: Development of Virtual Reality Health Literacy: Delphi Expert Consensus Study
Source: J Med Internet Res. 2026 Jun 24;28:e85842. doi: 10.2196/85842 (PMC13293473; doi:10.2196/85842)
Supplement: Multimedia Appendix 2 [file jmir-v28-e85842-s002.docx]

**Table S1.** Expert perspectives on definition of VR health literacy

| **Number of experts** | **Definition** |
| --- | --- |
| 1 | The ability to analyze and utilize health data obtained through VR. |
| 2 | Understanding that XR devices (AR/VR/MR) can provide health-related content and services beyond what PCs or smartphones offer and acquiring the capacity to act on this information to lead a healthier life. |
| 3 | While traditional literacy refers to basic reading comprehension, digital literacy also encompasses the ability to use technology and ethical attitudes toward its use. Similarly, VR health literacy involves the capacity to manage physical and mental health through metaverse-based content in virtual environments. |
| 4 | The ability to comprehend and accept health or educational information delivered through head-mounted displays (HMDs). |
| 5 | The capacity to use VR devices that display quantifiable health indicators, interpret the data, and formulate future health management plans accordingly. |
| 6 | The perception that embedding health information in VR makes it easier, clearer, and less burdensome to understand. |
| 7 | Providing immersive information environments in virtual spaces for general well-being, not necessarily limited to disease contexts, is a core function of digital healthcare. |
| 8 | Acquiring and applying health-related knowledge through VR. |
| 9 | Understanding and effectively using VR devices and services during health-related activities. |
| 10 | Due to the growth of digital therapeutics, there is increasing interest in VR-based health applications. VR health literacy refers to the ability to use VR devices in healthcare settings. |
| 11 | Whereas general health literacy involves maintaining health and preventing disease through informed decisions, VR health literacy emphasizes acquiring, understanding, and independently applying health information using VR. |
| 12 | VR (Virtual Reality) health literacy refers to the ability to manage and understand health through immersive VR technology. This includes gaining experience in physical activity and fitness, comprehending one’s health status, and using smart or wearable devices to make informed decisions. |
| 13 | Using virtual reality technology in the health domain requires knowledge, skills, and abilities related to safety and health. VR is currently applied in various fields, including medical diagnosis, treatment, training, and stress reduction. |
